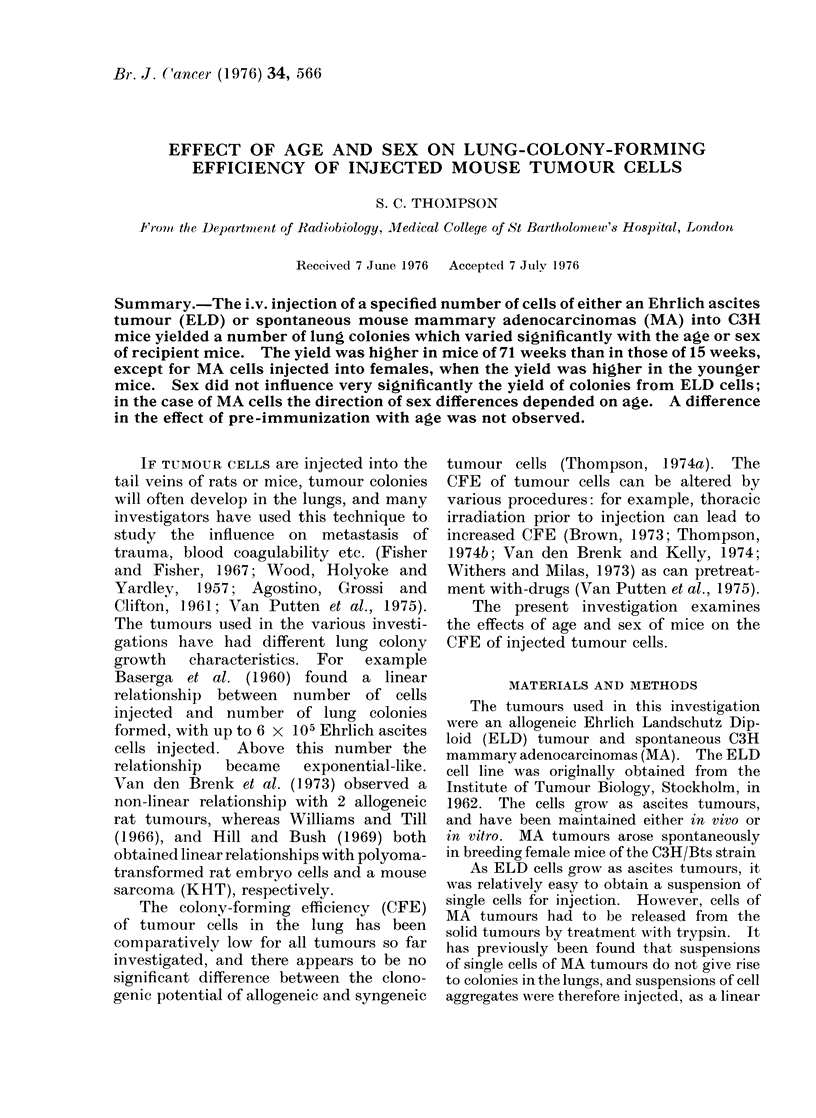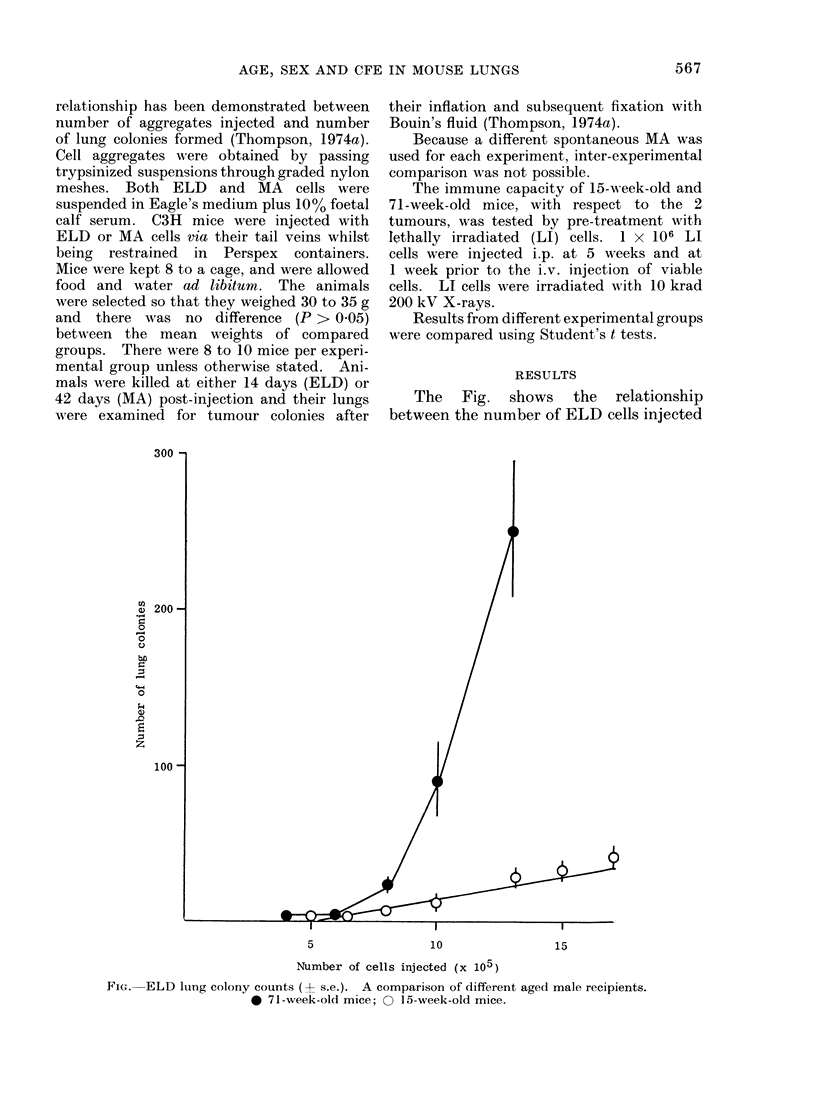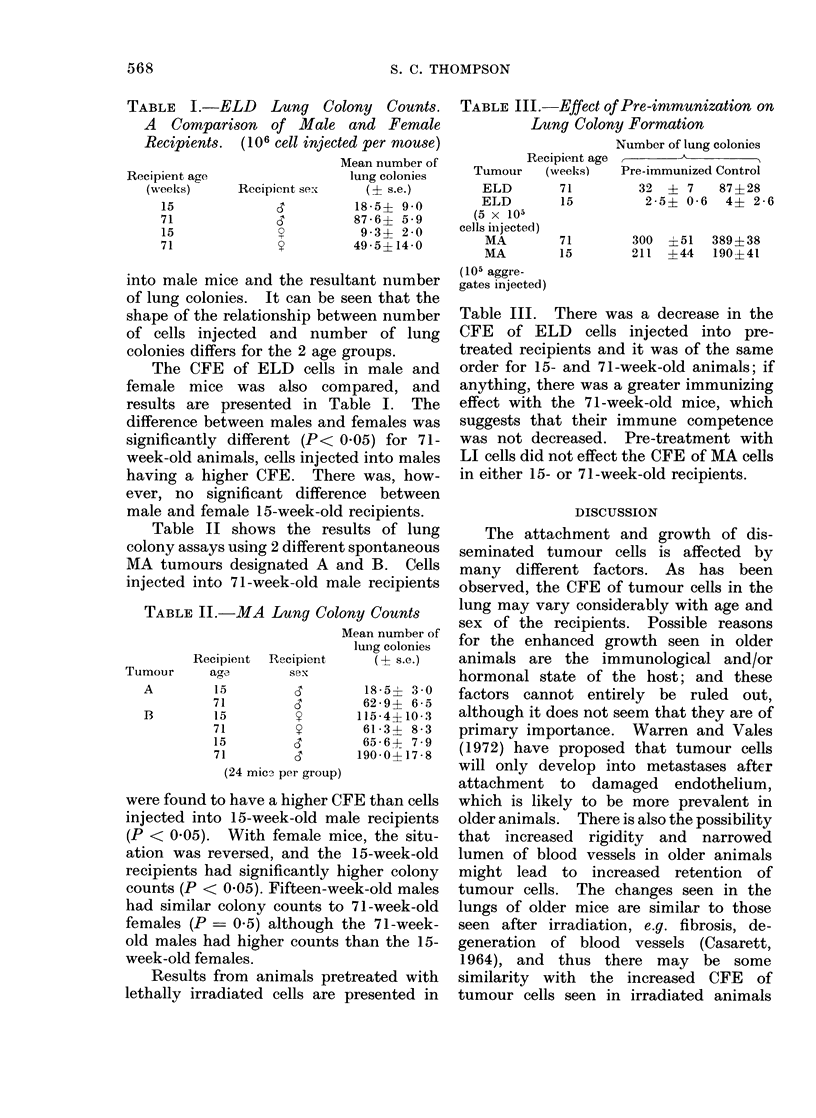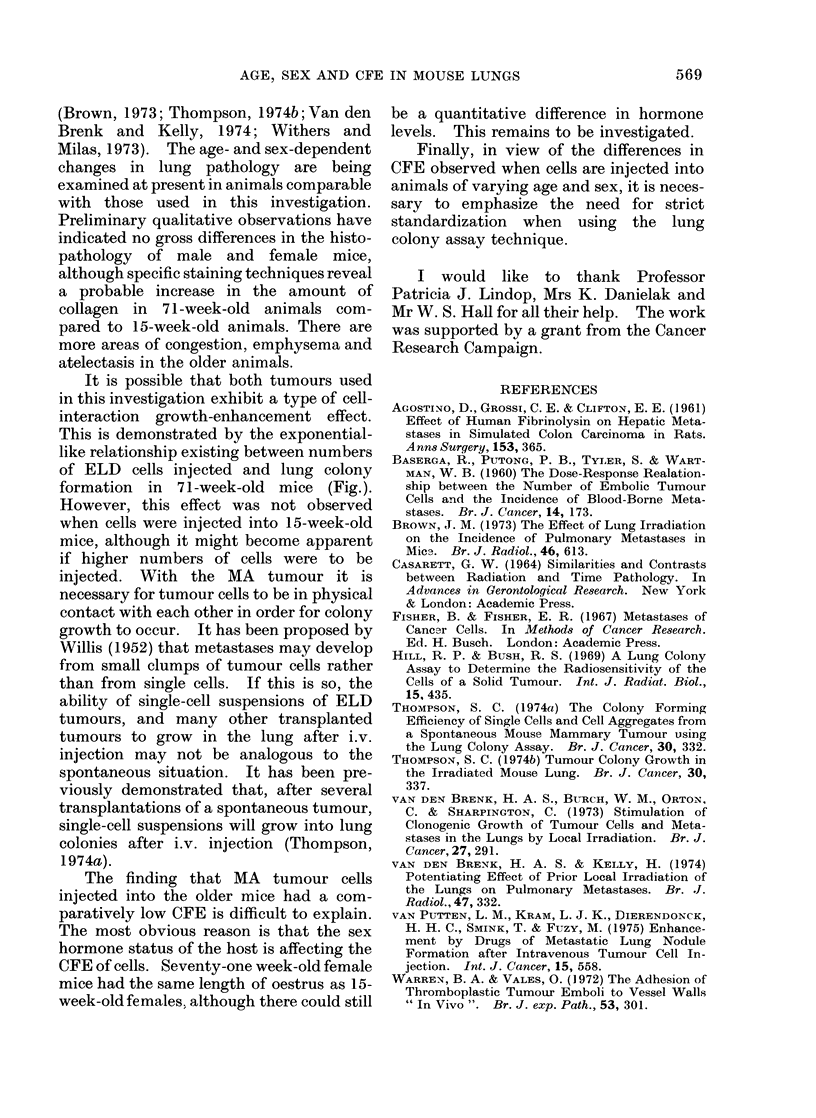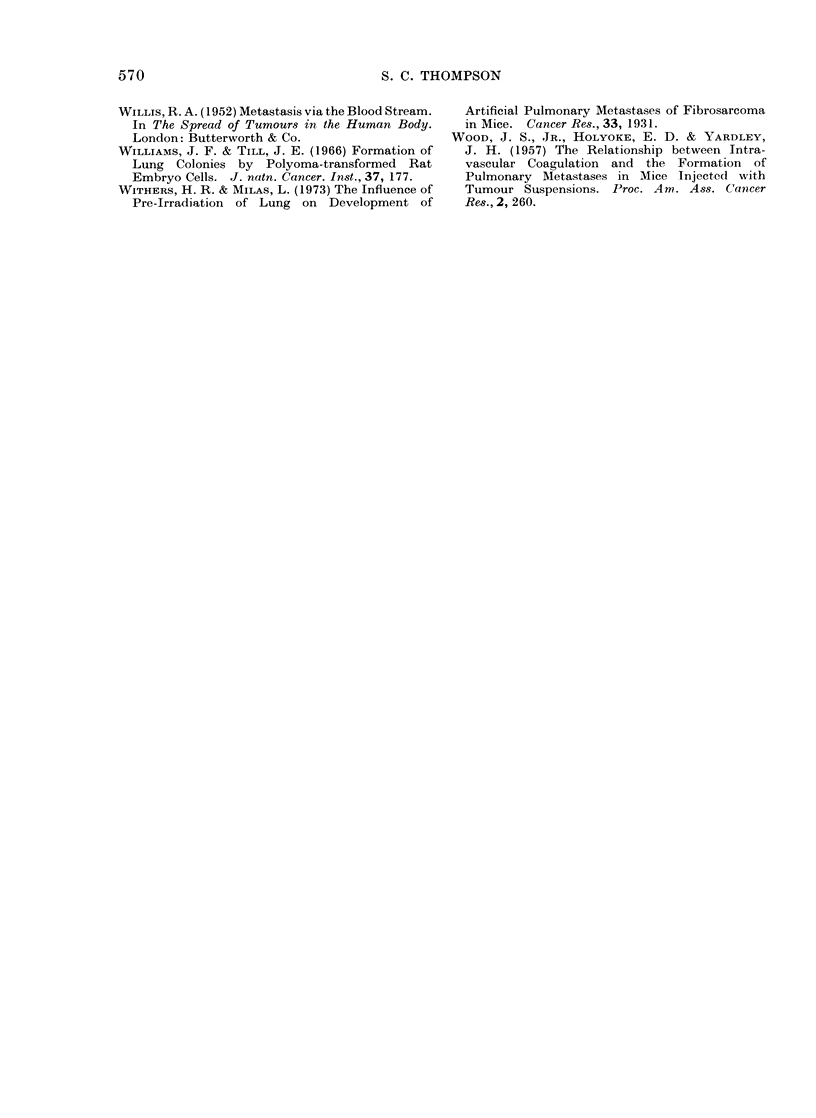# Effect of age and sex on lung-colony-forming efficiency of injected mouse tumour cells.

**DOI:** 10.1038/bjc.1976.212

**Published:** 1976-11

**Authors:** S. C. Thompson

## Abstract

The i.v. injection of a specified number of cells of either an Ehrlich ascites tumour (ELD) or spontaneous mouse mammary adenocarcinomas (MA) into C3H mice yielded a number of lung colonies which varied significantly with the age or sex of recipient mice. The yield was higher in mice of 71 weeks than in those of 15 weeks, except for MA cells injected into females, when the yield was higher in the younger mice. Sex did not influence very significantly the yield of colonies from ELD cells; in the case of MA cells the direction of sex differences depended on age. A difference in the effect of pre-immunization with age was not observed.


*Br. J. Cancer* (1976) **34**, 566

### EFFECT OF AGE AND SEX ON LUNG-COLONY-FORMING EFFICIENCY OF INJECTED MOUSE TUMOUR CELLS

S. C. THOMPSON

*From the Department of Radiobiology, Medical College of St Bartholomew's Hospital, London*

Received 7 June 1976 Accepted 7 July 1976

**Summary.—The i.v. injection of a specified number of cells of either an Ehrlich ascites tumour (ELD) or spontaneous mouse mammary adenocarcinomas (MA) into C3H mice yielded a number of lung colonies which varied significantly with the age or sex of recipient mice. The yield was higher in mice of 71 weeks than in those of 15 weeks, except for MA cells injected into females, when the yield was higher in the younger mice. Sex did not influence very significantly the yield of colonies from ELD cells; in the case of MA cells the direction of sex differences depended on age. A difference in the effect of pre-immunization with age was not observed.**

IF TUMOUR CELLS are injected into the tail veins of rats or mice, tumour colonies will often develop in the lungs, and many investigators have used this technique to study the influence on metastasis of trauma, blood coagulability etc. (Fisher and Fisher, 1967; Wood, Holyoke and Yardley, 1957; Agostino, Grossi and Clifton, 1961; Van Putten *et al.*, 1975). The tumours used in the various investigations have had different lung colony growth characteristics. For example Baserga *et al.* (1960) found a linear relationship between number of cells injected and number of lung colonies formed, with up to $6 \times 10^5$ Ehrlich ascites cells injected. Above this number the relationship became exponential-like. Van den Brenk *et al.* (1973) observed a non-linear relationship with 2 allogeneic rat tumours, whereas Williams and Till (1966), and Hill and Bush (1969) both obtained linear relationships with polyoma-transformed rat embryo cells and a mouse sarcoma (KHT), respectively.

The colony-forming efficiency (CFE) of tumour cells in the lung has been comparatively low for all tumours so far investigated, and there appears to be no significant difference between the clonogenic potential of allogeneic and syngeneic tumour cells (Thompson, 1974*a*). The CFE of tumour cells can be altered by various procedures: for example, thoracic irradiation prior to injection can lead to increased CFE (Brown, 1973; Thompson, 1974*b*; Van den Brenk and Kelly, 1974; Withers and Milas, 1973) as can pretreatment with-drugs (Van Putten *et al.*, 1975).

The present investigation examines the effects of age and sex of mice on the CFE of injected tumour cells.

### MATERIALS AND METHODS

The tumours used in this investigation were an allogeneic Ehrlich Landschutz Diploid (ELD) tumour and spontaneous C3H mammary adenocarcinomas (MA). The ELD cell line was originally obtained from the Institute of Tumour Biology, Stockholm, in 1962. The cells grow as ascites tumours, and have been maintained either *in vivo* or *in vitro*. MA tumours arose spontaneously in breeding female mice of the C3H/Bts strain

As ELD cells grow as ascites tumours, it was relatively easy to obtain a suspension of single cells for injection. However, cells of MA tumours had to be released from the solid tumours by treatment with trypsin. It has previously been found that suspensions of single cells of MA tumours do not give rise to colonies in the lungs, and suspensions of cell aggregates were therefore injected, as a linear relationship has been demonstrated between number of aggregates injected and number of lung colonies formed (Thompson, 1974*a*). Cell aggregates were obtained by passing trypsinized suspensions through graded nylon meshes. Both ELD and MA cells were suspended in Eagle's medium plus 10% foetal calf serum. C3H mice were injected with ELD or MA cells *via* their tail veins whilst being restrained in Perspex containers. Mice were kept 8 to a cage, and were allowed food and water *ad libitum*. The animals were selected so that they weighed 30 to 35 g and there was no difference ($P > 0{\cdot}05$) between the mean weights of compared groups. There were 8 to 10 mice per experimental group unless otherwise stated. Animals were killed at either 14 days (ELD) or 42 days (MA) post-injection and their lungs were examined for tumour colonies after their inflation and subsequent fixation with Bouin's fluid (Thompson, 1974*a*).

Because a different spontaneous MA was used for each experiment, inter-experimental comparison was not possible.

The immune capacity of 15-week-old and 71-week-old mice, with respect to the 2 tumours, was tested by pre-treatment with lethally irradiated (LI) cells. $1 \times 10^6$ LI cells were injected i.p. at 5 weeks and at 1 week prior to the i.v. injection of viable cells. LI cells were irradiated with 10 krad 200 kV X-rays.

Results from different experimental groups were compared using Student's *t* tests.

### RESULTS

The Fig. shows the relationship between the number of ELD cells injected

FIG.—ELD lung colony counts (± s.e.). A comparison of different aged male recipients.
● 71-week-old mice; ○ 15-week-old mice.

TABLE I.—*ELD Lung Colony Counts. A Comparison of Male and Female Recipients.* ($10^6$ *cell injected per mouse*)

| Recipient age (weeks) | Recipient sex | Mean number of lung colonies (± s.e.) |
|---|---|---|
| 15 | ♂ | 18·5± 9·0 |
| 71 | ♂ | 87·6± 5·9 |
| 15 | ♀ | 9·3± 2·0 |
| 71 | ♀ | 49·5±14·0 |

into male mice and the resultant number of lung colonies. It can be seen that the shape of the relationship between number of cells injected and number of lung colonies differs for the 2 age groups.

The CFE of ELD cells in male and female mice was also compared, and results are presented in Table I. The difference between males and females was significantly different ($P < 0·05$) for 71-week-old animals, cells injected into males having a higher CFE. There was, however, no significant difference between male and female 15-week-old recipients.

Table II shows the results of lung colony assays using 2 different spontaneous MA tumours designated A and B. Cells injected into 71-week-old male recipients

TABLE II.—*MA Lung Colony Counts*

| Tumour | Recipient age | Recipient sex | Mean number of lung colonies (± s.e.) |
|---|---|---|---|
| A | 15 | ♂ | 18·5± 3·0 |
| | 71 | ♂ | 62·9± 6·5 |
| B | 15 | ♀ | 115·4±10·3 |
| | 71 | ♀ | 61·3± 8·3 |
| | 15 | ♂ | 65·6± 7·9 |
| | 71 | ♂ | 190·0±17·8 |

(24 mice per group)

were found to have a higher CFE than cells injected into 15-week-old male recipients ($P < 0·05$). With female mice, the situation was reversed, and the 15-week-old recipients had significantly higher colony counts ($P < 0·05$). Fifteen-week-old males had similar colony counts to 71-week-old females ($P = 0·5$) although the 71-week-old males had higher counts than the 15-week-old females.

Results from animals pretreated with lethally irradiated cells are presented in Table III. There was a decrease in the CFE of ELD cells injected into pre-treated recipients and it was of the same order for 15- and 71-week-old animals; if anything, there was a greater immunizing effect with the 71-week-old mice, which suggests that their immune competence was not decreased. Pre-treatment with LI cells did not effect the CFE of MA cells in either 15- or 71-week-old recipients.

TABLE III.—*Effect of Pre-immunization on Lung Colony Formation*

| Tumour | Recipient age (weeks) | Number of lung colonies: Pre-immunized | Control |
|---|---|---|---|
| ELD | 71 | 32 ± 7 | 87±28 |
| ELD | 15 | 2·5± 0·6 | 4± 2·6 |
| ($5 \times 10^5$ cells injected) | | | |
| MA | 71 | 300 ±51 | 389±38 |
| MA | 15 | 211 ±44 | 190±41 |
| ($10^5$ aggregates injected) | | | |

### DISCUSSION

The attachment and growth of disseminated tumour cells is affected by many different factors. As has been observed, the CFE of tumour cells in the lung may vary considerably with age and sex of the recipients. Possible reasons for the enhanced growth seen in older animals are the immunological and/or hormonal state of the host; and these factors cannot entirely be ruled out, although it does not seem that they are of primary importance. Warren and Vales (1972) have proposed that tumour cells will only develop into metastases after attachment to damaged endothelium, which is likely to be more prevalent in older animals. There is also the possibility that increased rigidity and narrowed lumen of blood vessels in older animals might lead to increased retention of tumour cells. The changes seen in the lungs of older mice are similar to those seen after irradiation, *e.g.* fibrosis, degeneration of blood vessels (Casarett, 1964), and thus there may be some similarity with the increased CFE of tumour cells seen in irradiated animals

(Brown, 1973; Thompson, 1974*b*; Van den Brenk and Kelly, 1974; Withers and Milas, 1973). The age- and sex-dependent changes in lung pathology are being examined at present in animals comparable with those used in this investigation. Preliminary qualitative observations have indicated no gross differences in the histopathology of male and female mice, although specific staining techniques reveal a probable increase in the amount of collagen in 71-week-old animals compared to 15-week-old animals. There are more areas of congestion, emphysema and atelectasis in the older animals.

It is possible that both tumours used in this investigation exhibit a type of cell-interaction growth-enhancement effect. This is demonstrated by the exponential-like relationship existing between numbers of ELD cells injected and lung colony formation in 71-week-old mice (Fig.). However, this effect was not observed when cells were injected into 15-week-old mice, although it might become apparent if higher numbers of cells were to be injected. With the MA tumour it is necessary for tumour cells to be in physical contact with each other in order for colony growth to occur. It has been proposed by Willis (1952) that metastases may develop from small clumps of tumour cells rather than from single cells. If this is so, the ability of single-cell suspensions of ELD tumours, and many other transplanted tumours to grow in the lung after i.v. injection may not be analogous to the spontaneous situation. It has been previously demonstrated that, after several transplantations of a spontaneous tumour, single-cell suspensions will grow into lung colonies after i.v. injection (Thompson, 1974*a*).

The finding that MA tumour cells injected into the older mice had a comparatively low CFE is difficult to explain. The most obvious reason is that the sex hormone status of the host is affecting the CFE of cells. Seventy-one week-old female mice had the same length of oestrus as 15-week-old females, although there could still be a quantitative difference in hormone levels. This remains to be investigated.

Finally, in view of the differences in CFE observed when cells are injected into animals of varying age and sex, it is necessary to emphasize the need for strict standardization when using the lung colony assay technique.

I would like to thank Professor Patricia J. Lindop, Mrs K. Danielak and Mr W. S. Hall for all their help. The work was supported by a grant from the Cancer Research Campaign.

REFERENCES

AGOSTINO, D., GROSSI, C. E. & CLIFTON, E. E. (1961) Effect of Human Fibrinolysin on Hepatic Metastases in Simulated Colon Carcinoma in Rats. *Anns Surgery*, **153**, 365.

BASERGA, R., PUTONG, P. B., TYLER, S. & WARTMAN, W. B. (1960) The Dose-Response Realationship between the Number of Embolic Tumour Cells and the Incidence of Blood-Borne Metastases. *Br. J. Cancer*, **14**, 173.

BROWN, J. M. (1973) The Effect of Lung Irradiation on the Incidence of Pulmonary Metastases in Mice. *Br. J. Radiol.*, **46**, 613.

CASARETT, G. W. (1964) Similarities and Contrasts between Radiation and Time Pathology. In *Advances in Gerontological Research.* New York & London: Academic Press.

FISHER, B. & FISHER, E. R. (1967) Metastases of Cancer Cells. In *Methods of Cancer Research.* Ed. H. Busch. London: Academic Press.

HILL, R. P. & BUSH, R. S. (1969) A Lung Colony Assay to Determine the Radiosensitivity of the Cells of a Solid Tumour. *Int. J. Radiat. Biol.*, **15**, 435.

THOMPSON, S. C. (1974*a*) The Colony Forming Efficiency of Single Cells and Cell Aggregates from a Spontaneous Mouse Mammary Tumour using the Lung Colony Assay. *Br. J. Cancer*, **30**, 332.

THOMPSON, S. C. (1974*b*) Tumour Colony Growth in the Irradiated Mouse Lung. *Br. J. Cancer*, **30**, 337.

VAN DEN BRENK, H. A. S., BURCH, W. M., ORTON, C. & SHARPINGTON, C. (1973) Stimulation of Clonogenic Growth of Tumour Cells and Metastases in the Lungs by Local Irradiation. *Br. J. Cancer*, **27**, 291.

VAN DEN BRENK, H. A. S. & KELLY, H. (1974) Potentiating Effect of Prior Local Irradiation of the Lungs on Pulmonary Metastases. *Br. J. Radiol.*, **47**, 332.

VAN PUTTEN, L. M., KRAM, L. J. K., DIERENDONCK, H. H. C., SMINK, T. & FUZY, M. (1975) Enhancement by Drugs of Metastatic Lung Nodule Formation after Intravenous Tumour Cell Injection. *Int. J. Cancer*, **15**, 558.

WARREN, B. A. & VALES, O. (1972) The Adhesion of Thromboplastic Tumour Emboli to Vessel Walls " In Vivo ". *Br. J. exp. Path.*, **53**, 301.

WILLIS, R. A. (1952) Metastasis via the Blood Stream. In *The Spread of Tumours in the Human Body*. London: Butterworth & Co.

WILLIAMS, J. F. & TILL, J. E. (1966) Formation of Lung Colonies by Polyoma-transformed Rat Embryo Cells. *J. natn. Cancer. Inst.*, **37**, 177.

WITHERS, H. R. & MILAS, L. (1973) The Influence of Pre-Irradiation of Lung on Development of Artificial Pulmonary Metastases of Fibrosarcoma in Mice. *Cancer Res.*, **33**, 1931.

WOOD, J. S., JR., HOLYOKE, E. D. & YARDLEY, J. H. (1957) The Relationship between Intravascular Coagulation and the Formation of Pulmonary Metastases in Mice Injected with Tumour Suspensions. *Proc. Am. Ass. Cancer Res.*, **2**, 260.